# Mechanical–Chemical Activation of Cement-Ash Binders to Improve the Properties of Heat-Resistant Mortars

**DOI:** 10.3390/ma17235760

**Published:** 2024-11-25

**Authors:** Leonid Dvorkin, Vadim Zhitkovsky, Tomasz Tracz, Mateusz Sitarz, Katarzyna Mróz

**Affiliations:** 1 Department of Building Elements Technology and Materials Science, National University of Water and Environmental Engineering, 33028 Rivne, Ukraine; l.i.dvorkin@nuwm.edu.ua; 2 Chair of Building Materials Engineering, Faculty of Civil Engineering, Cracow University of Technology, 31-155 Cracow, Poland; tomasz.tracz@pk.edu.pl (T.T.); katarzyna.mroz@pk.edu.pl (K.M.)

**Keywords:** cement-ash binder, heat resistance, activation, admixture, sodium fluorosilicate, experimental–statistical model, thermal deformation

## Abstract

The article demonstrates the effectiveness of the mechanochemical activation of a cement-ash binder by increasing the specific surface area of the ash and introducing a sodium fluorosilicate additive (Na_2_SiF_6_). It has been experimentally proved that the introduction of a Na_2_SiF_6_ additive makes it possible to increase the degree of cement hydration, as well as the intensity of free CaO binding when heating the cement-ash binder in the range of 500 °C to 800 °C. Mechanochemical activation prevents a decrease in the strength of the preheated cement-ash binder. During cyclic heating and cooling of slag mortars based on the activated cement-ash binder, an improvement in the set of basic properties was observed: compressive strength, flexural strength, water absorption, dynamic modulus of elasticity, and conditional elongation. Experimental design was carried out to obtain experimental–statistical models of mortar properties based on composition, heating temperature, and number of heating–cooling cycles. These models made it possible to develop quantitative relationships for predicting mortar properties at elevated temperatures and to rank the factors in order of importance. The optimal values for the dosage of fly ash, sodium silicofluoride additive, and the binder’s specific surface area were established. It was demonstrated that the activator has a positive effect on the thermal deformation of mortars.

## 1. Introduction

Portland cement-based concretes and mortars are used at service temperatures of up to 200 °C. It is known that the heat resistance of Portland cement can be increased by introducing active mineral additives into its composition or directly into the concrete mix [1].

Some researchers [2,3,4,5,6] have found that finely ground additives:

- bind free calcium oxide formed during the decomposition of hydrated compounds during heating, thereby eliminating the possibility of its quenching;
- do not form easily melting substances with Portland cement minerals;
- are resistant to high temperatures;
- reduce the shrinkage of hydrated Portland cement when heated;
- do not reduce the activity of Portland cement.

The work of many researchers has shown that free calcium oxide binds well at high temperatures when interacting with materials containing active silica and alumina [7,8,9,10]. The interaction reaction between amorphous silica and calcium oxide in the solid state is intensive at 500–600 °C [10].

Research has shown that finely ground additives can be a variety of siliceous and alumino-siliceous materials such as fireclay, chromite, magnesite, fly ash, granulated blast furnace fuel slag, etc. [4,8]. The choice of the additive type is influenced by the possible service temperature of the concrete. In heat-resistant concrete, which is intended for use at 800–900 °C, it is impractical to introduce expensive and scarce finely ground additives of high refractoriness, such as chromite, magnesite, etc. Fly ash is one of the most common active mineral additives in cement mortars and concrete. The main component of fly ash that determines its activity is the vitreous aluminosilicate phase, which makes up 40–65% of its total mass and contains spherical particles up to 100 μm in size [11,12,13,14,15,16,17].

The analysis of studies of the processes of structure formation of cement systems allows us to assume that the activity of mineral additives, including ash, is characterized by their ability to exert both chemical and physicochemical influences on the processes of artificial stone formation, which is important under the influence of both normal and elevated temperatures. The chemical activity of mineral additives is, in most cases, of a pozzolanic nature and is sufficiently well studied. The ability of active mineral additives to participate in the physical and chemical processes of organizing the structure of cement stone, mortars, and concretes has been studied to a much lesser extent.

The activation of mineral additives is understood as a set of technological methods aimed at increasing their activity [18]. Activation methods have mostly been developed for cement. The main ones are grinding, vibration activation, turbulent, acoustic, ultrasonic, thermal, aerothermal, and electric pulse processing [19,20]. Some recommendations have also been developed for the activation of binders and mineral additives by surface modification with various chemical substances, including surfactants, halogenated, alkaline, and organosilicon substances [9,21,22].

The essence of mechanical activation is to increase the reactivity of powders through the opening of new active surfaces of grains, changes in the crystal structure of minerals, the formation of a large number of unsaturated valence bonds, and deep grinding and their amorphization. The most remarkable possibilities for the activation of cement-ash systems are manifested when they are finely ground in the presence of superplasticizers introduced either during grinding or during subsequent mixing [9,20,21,22,23,24].

It has been established that the activation of hydration and the increase in strength of cement binders is achieved by introducing substances into the binding system, mainly ionic substances due to the chemical nature of the bond, as well as substances with oxidizing properties [21]. This method of activation is most effective for low-reactive cement with the introduction of ash and slag materials. The effectiveness of introducing additives of fluoride salts (CaF_2_, MgF_2_, NaF) with oxidizing agents—potassium permanganate and chromate with sodium sulfide—on the strength of various slag binders was studied. It was also found that the use of activators made it possible to obtain a clinker-free binder with strength at 28 days of up to 30 MPa. At the same time, the strength of the binders at the early 1 and 3 days of hardening increased significantly.

According to theoretical ideas [21,24], substances containing fluorine ions can act in three directions:

- by activating the breaking of Si-O bonds and the transfer of silicon ions into the mortar;
- by acting on the surface of minerals, replacing the OH group;
- as replacement of an oxygen atom by F on the surface of cement minerals.

When activated by fluoride salts, not only is chemical modification of the surface achieved but also control of surface electronic processes.

In Portland cement-based concretes, fluoride salts can be considered activators that not only intensify the structure formation processes at normal temperatures but also contribute to the interaction of CaO, with the aluminosilicate component at elevated temperatures acting as mineralizers. The role of an aluminosilicate component that binds CaO, together with other finely ground additives, can be performed by ash. The use of fly ash, as well as other mineral additives, improves the thermal resistance of cement concrete. While this has been demonstrated in various studies, the improvement in thermal resistance when using fly ash alone remains modest and requires further optimization.

At present, the combined effect of the mechanical activation of cement-ash binders and their chemical activation with the addition of superplasticizers and electrolytes, in particular fluoride salts, on the physical and mechanical properties of cement both at normal and elevated temperatures requires special research. This article presents the results of the influence of the mechanochemical activation of cement-ash binders on the main properties of cement-ash binders and heat-resistant concretes based on them in the temperature range between 300 °C and 800 °C. The paper presents an original and novel approach that involves an increase in the fineness of the binder’s grinding with the addition of sodium silicofluoride. The method has been demonstrated to significantly enhance the thermal stability of cement-ash binders.

## 2. Research Materials and Methods

The raw materials used in the research were Portland cement CEM-II/A-S from the Ivano-Frankivsk plant and fly ash from the Burshtyn TPP (Ukraine). Naphthalene-formaldehyde superplasticizer “Polyplast SP-1” and sodium fluorosilicate Na_2_SiF_6_ were used as chemical additives.

The chemical and mineralogical composition of Portland cement and its physical and mechanical properties are given in Table 1 and Table 2. The chemical composition of the cement was obtained using the standard method [25]. The mineralogical composition was calculated based on chemical analysis data using a standardized methodology [25].

The chemical composition of the applied fly ash, its specific surface area, as well as its physical properties are given below:

- chemical composition: A1_2_O_3_—21.5%; SO_3_—2.3%, free CaO (CaO_f_)—0.8%, MgO—2.1%, Na_2_O + K_2_O—1.1%, L.O.I.—4.30%;
- specific surface area—2800 cm^2^/g;
- physical properties of the fly ash: true density—2220 kg/m^3^; bulk density—900 kg/m^3^; normal consistency—26.7%; humidity—1.4%; activity on absorption CaO—39.4 mg/g.

The composition and properties of Portland cement were determined according to [26], and fly ash—according to [27].

Sodium-silicon fluoride with a Na_2_SiF_6_ content of at least 95% was used as a fluoride activator for the cement-ash binder.

Fuel slag with a fineness modulus of 3.4 and chemical composition (SiO_2_—41.6%; A1_2_O_3_—12.5%; CaO—32.4%; Fe_2_O_3_—11.8%) was used as an aggregate for heat-resistant concrete. The content of unburnt fuel was 1.7%.

**Figure A1:**
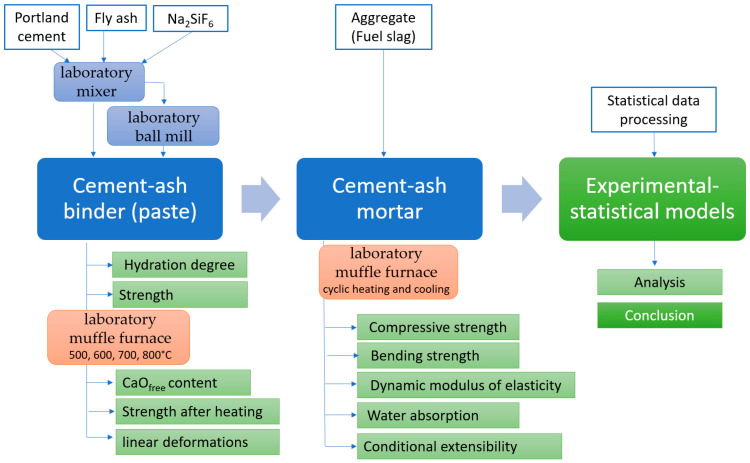
Flowchart of the conducted research.

The flowchart for conducting the research is shown in Appendix A, Figure A1.

Grinding of the binder with the addition of ash was carried out under cyclic loading in a laboratory ball mill. Standard samples of cement-ash binder and concrete were heated in a laboratory muffle furnace.

The specific surface area of the cement-ash binder was determined by measuring its air permeability (according to Blaine) [28].

To determine the influence of ash and the activating additive, Na_2_SiF_6_, on the degree of hydration of cement-ash binders, the content of chemically bound water was determined. For this purpose, 20 mm × 20 mm × 20 mm cube samples were prepared from cement and cement-ash mixes of normal consistency and stored in a desiccator with water until the time of testing (temperature 20 ± 2 °C, humidity 90–100%). After the specified time to remove mechanically bound water and stop the hydration process, the samples were crushed and treated with absolute ethyl alcohol and then calcined to a constant mass [25]. Free calcium oxide was determined by the ethylene–glycerate method based on the extraction of calcium oxide from a freshly ground powder of the analyzed sample with ethanol–glycerin, followed by titration of the resulting calcium glycerate with anhydrous benzoic acid in the presence of phenolphthalein [29,30].

The temperature deformations of the cement-ash paste were determined on a tripod with a clock-type indicator.

Cyclic heating of dried mortar samples of beams with dimensions 40 × 40 × 160 mm at a ratio of cement-ash binder: aggregate 1:3 was carried out in a muffle furnace with a temperature rise rate of 150 °C/h with exposure at a given temperature for 4 h and cooling to 20 °C.

The strength of mortars was determined according to [31]. Water absorption was calculated according to Formula (1).

(1) $$W_{ab}=\frac{m_{w}-m_{dry}}{m_{dry}}\times 100,$$

where

*m_w_*—is the mass of the sample after saturation with water, g;

*m_dry_*—is the mass of the dry sample, g.

The mortar dynamic modulus of elasticity *E_d_* was determined by the resonance method [32,33,34,35] on a laboratory setup consisting of a generator of sound frequencies of the exciter and a receiver of mechanical vibrations and was calculated according to Formula (2).

(2) $$E_{d}=0.4l^{2}f_{l}^{2}\rho ,$$

where

*l*—sample length;

*f_l_*—frequency of longitudinal oscillations;

*ρ*—density.

The conditional elongation of the mortars was calculated according to Formula (3).

(3) $$\varepsilon_{c}=\frac{f_{b}}{E_{d}}$$

where

*f_b_*—bending strength of the sample, MPa.

The influence of a complex of factors determining the degree and efficiency of activation of the cement-ash binder on the strength and properties of heat-resistant concrete was studied using a method of experimental design [36]. This method allows experiments to be conducted using an optimal design matrix and statistical processing of test results to obtain accurate experimental–statistical models in the form of linear dependencies; Formula (4).

(4) $$y=b_{0}+\sum_{i=1}^{k}b_{i}x_{i}+\sum_{i=1}^{k}b_{ij}x_{i}x_{j},$$

where

*y*—is the initial parameter;

*b*_0_, *b_i_*, *b_ij_*—are the regression coefficients;

*x_i_*, *x_ij_*—are the investigated factors;

*k*—is the number of factors.

The regression coefficient values provide information regarding the effect of appropriate factors on the initial parameter or property.

The experiments followed a two-stage 2^4−1^ design plan [37] containing 8 experimental points (Table 3 and Table 4).

The algebraically calculated quantitative assessments of the coefficients of the equations were subjected to statistical analysis [37]. At the first stage of regression analysis, the standard deviation of the initial parameter and mean quadratic errors of models’ estimation coefficients are obtained. The coefficients are valuable if the design value of the Student’s t-criterion is more than the given one. If a coefficient is not important, it can be omitted without re-calculating other coefficients. After the importance of the coefficients is estimated, the equation’s adequacy is checked by calculating the adequacy dispersion, the design value of Fischer’s criterion (F—criterion) (F_c_), and comparing the last with a given one. The given value of the F—criterion (F_t_) is obtained depending on the confidence probability (importance level) of 95% and the number of degrees of freedom. The equation is adequate for the given probability level if F_c_ > F_t_. This process was carried out using a computer program called PPP. The results of the calculation of the statistical characteristics are given in Appendix B, Table A1.

## 3. Results and Discussion

The results of determining the effect of the Na_2_SiF_6_ activator on the strength of a standard Portland cement mortar with ash addition during setting under normal conditions are given in Table 5.

As can be seen from Table 5, grinding and the addition of 0.5% Na_2_SiF_6_ by weight of the binder helps to increase the compressive strength at a 30% content of fly ash (Table 5) and hardening under normal conditions after 1 day by 49%; 1% by 63%; after 3 days the increase in compressive strength is 40–44%, respectively. Flexural strength also increases within these limits.

The degrees of cement hydration in the tested samples are given in Table 6.

From the data obtained, it can be concluded that the introduction of fly ash into the cement without changing the specific surface (3500 cm^2^/g) in the amount of 20–30% reduces the degree of hydration at an early age (up to 3 days) in the range of 10–20%. When the specific surface is increased to 4200 cm^2^/g, no decrease in the hydration degree is observed both at the age of 28 days and at an earlier age, starting from the age of 1 day of the cement-ash binder. The introduction of a 1% Na_2_SiF_6_ additive increases the hydration degree of cement-ash stone both at an early age and at a later age.

**Figure 1:**
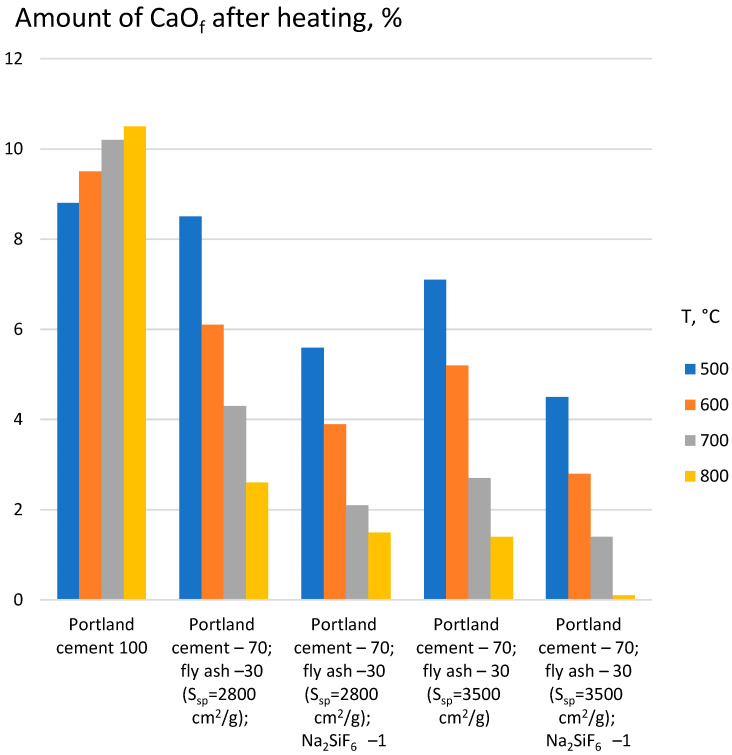
The influence of the composition and fineness of grinding of the cement-ash binder on CaO_free_ (CaO_f_) content after heating.

To study the process of formation and binding of CaO during heating of the cement-ash binder with the Na_2_SiF_6_ additive, a sample of dough of normal consistency, after hardening for 3 days under normal conditions, was heated in a laboratory muffle furnace to temperatures of 500, 600, 700, and 800 °C with a holding time of 1 h. The data from the chemical analysis show that the reaction of intense binding of CaO during heating of the cement-ash binder is observed in the range of 500 °C to 800 °C, with an intensity that depends on the dispersion of the ash and the presence of the Na_2_SiF_6_ additive (Table 7, Figure 1).

The introduction of a 1% Na_2_SiF_6_ additive makes this process noticeable already at 500 °C. The obtained experimental data show that the activating of the CaO absorption by ash and contributing to the formation of additional hydrosilicates and hydroaluminates in the range of 500 °C to 800 °C, the Na_2_SiF_6_ additive acts as a mineralizer, contributing to the intensification of solid-phase reactions into the CaO–ash system [15].

Sodium fluorosilicate is stable at temperatures up to 400 °C [15]. At a higher temperature, it decomposes into sodium fluoride and silicon fluoride according to the reaction: Na_2_SiF_6_ → 2NaF + SiF_4_. During heat treatment of a mixture of CaCO_3_ and NaF, a compound is formed, the composition of which corresponds to the formula CaO·3NaF. This compound catalytically participates in the reaction of the formation of calcium silicates, significantly accelerating it.

Table 8 shows the values of the relative compressive strength of samples of the cement-ash binder at the age of 28 days with W/C equal to the normal consistency and the kinetics of its change during heating.

**Figure 2:**
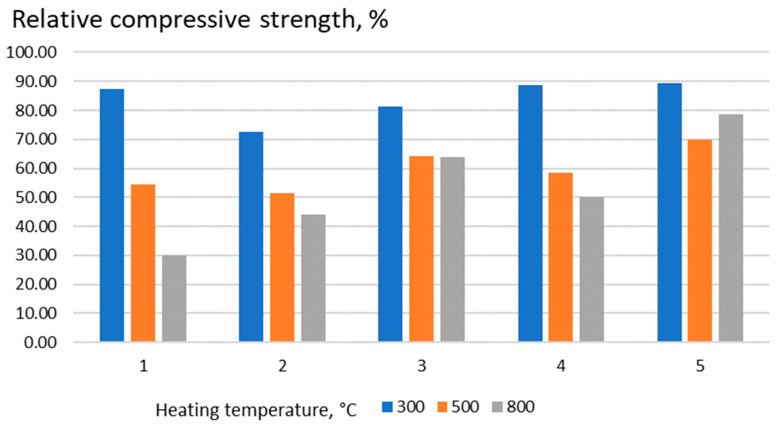
The influence of the composition and fineness of binder grinding on compressive strength. Positions according to Table 8.

As the temperature rises above 300 °C, the strength gradually decreases. The degree of an ultimate strength decrease at 800 °C is not the same for the binders under study. For a cement-ash binder, it is 44 to 50%, and with the addition of sodium silicofluoride, it is 63 to 78% of the initial strength of the binder before heating (Figure 2). Increasing the fineness of the ash grinding results in both an increase in the initial strength of the cement-ash paste and a more gradual decrease during heating.

Along with the change in the strength of the cement-ash binder during heating, the change in strength of the heated samples during further storage for 3 days was studied. The obtained results indicate that for samples of cement-ash heated at T ≤ 500 °C and during further storage in air-moist conditions for 3 days, the loss of strength is about 20%; for samples heated at T = 600 °C and 800 °C, the loss of strength reaches 80%. For cement-ash paste heated in the temperature range of 500 °C to 800 °C during further storage in normal air-moist conditions (temperature 20 ± 2 °C, relative humidity not less than 90%), the loss of strength was 0 to 20%. With increasing dispersion of the cement-ash binder, as well as with the introduction of the Na_2_SiF_6_ additive, the decrease in strength of the preheated specimens becomes limited. It correlates with the content of free calcium oxide.

For heat-resistant mortars, along with the ultimate strength at a possible permissible temperature, the amount of temperature shrinkage is also normalized [25].

The amount of thermal shrinkage of concrete is determined by the thermal shrinkage of the cement paste that occurs during the first heating. During repeated heating, some expansion is observed for cement stone both without finely ground additives and with additives [3].

The test results for linear deformation of the heated cement-ash paste are given in Table 9. These results show that when the ash filler is introduced, there is a slight decrease in the relative linear deformations. The presence of the Na_2_SiF_6_ binder in the composition reinforces the positive trends of the effect of the ash on the deformation of the cement paste during heating. This effect is due to an increase in the density of cement paste, which is especially important for ensuring the density of mortars at elevated temperatures.

The operational reliability of a heat-resistant mortar largely depends on ensuring its normalized properties during cyclic heating and cooling, which, as a result of destructive processes, causes the accumulation of internal stresses, the formation of microcracks, and a decrease in strength.

Experimental determination of cyclic heating and cooling of the mortar on the cement-ash binder was carried out using mathematical planning of the experiment. The conditions for the design of experiments are given in Table 3, while the matrixes and the results of the implementation of the experimental design are presented in Table 4 and Table 10. The variation intervals of the factors are chosen to cover the most probable compositions of concrete subjected to cyclic heating.

The beam specimens were made from mortar mixes based on a cement-ash binder using Portland cement (CEM 42.5 II, 20% granulated blast furnace slag, Ssp = 2900 cm^2^/g) and the introduction of fly ash (30% of the cement mass) during mixing.

To achieve the required workability of the mortar mixtures, the water consumption was considered according to the immersion depth of the cone of 10–40 mm for C/W = 1.5–215 L/m^3^ and C/W = 2.5–230 L/m^3^.

The design matrix and the results of the experiments are shown in Table 4 and Table 10.

Experimental–statistical models for indicators of properties of the mortars subjected to cyclic heating and cooling (Table 10), obtained as a result of the statistical processing of the experimental data (the designation of variables (X_1_–X_4_) corresponds to Table 3), are as follows:

Compressive strength (*f_c_*), MPa:

(5) $$f_{c}=11.237+3.662X_{1\;}+2.962X_{2}-0.83X_{4}+0.537X_{1}X_{2\;}-0.262X_{1}X_{3}$$

Bending strength (*f_b_*), MPa:

(6) $$f_{b}=2.225+0.55X_{1}+0.225X_{2\;}-0.05X_{3}-\;0.175X_{4}+0.05X_{1}X_{2}$$

Dynamic modulus of elasticity (*E_d_*), 10^4^ MPa:

(7) $$E_{d}=2.205+0.305X_{1\;}+0.28X_{2}-0.102X_{3}-\;0.172X_{4}-0.047X_{1}X_{3}+0.077X_{1}X_{4}$$

Water absorption (*W_ab_*),%:

(8) $$W_{ab}=11.35-1.275X_{1}-1.175X_{2}+0.3X_{4}-\;0.6X_{1}X_{2\;}-0.125X_{1}X_{4}$$

Conditional extensibility (*ε_c_*):

(9) $$\varepsilon_{c}=0.999+0.106X_{1}-0.025X_{2}-0.017X_{3}-\;0.004X_{4}+0.02X_{1}X_{2}-0.022X_{1}X_{4},$$

where

X_1_ is the content additive Na_2_SiF_6_ (D) (%);

X_2_ is the cement–water ratio, (C/W);

X_3_ is the heating temperature, (T) (°C);

X_4_ is the number of cycles of heating and cooling, (N).

**Figure 3:**
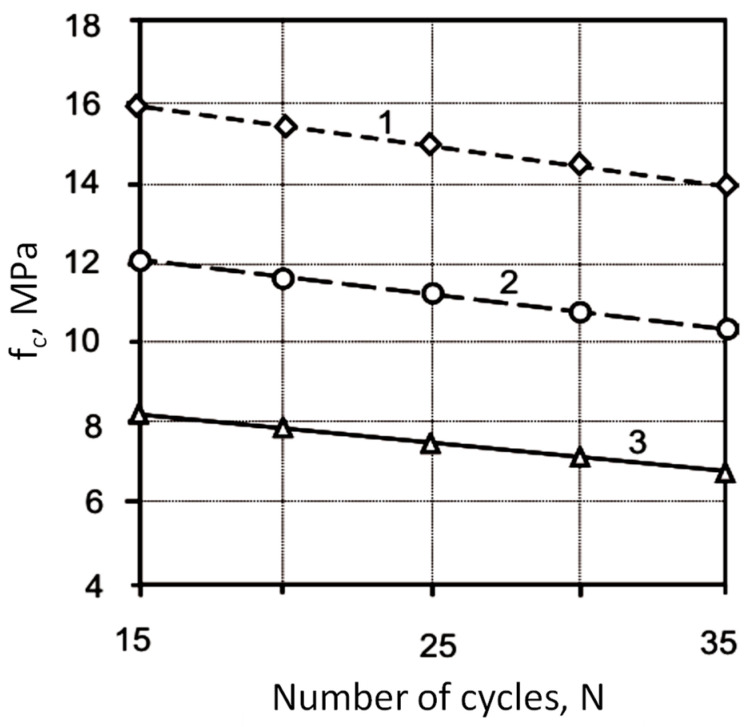
Influence of the number of heating and cooling cycles (*N*) and consumption of the additive (*D*) Na_2_SiF_6_ on the compressive strength (*f_c_*) of the mortars: 1—*D* = 1%; 2—*D* = 0.5%; 3—*D* = 0%.

**Figure 4:**
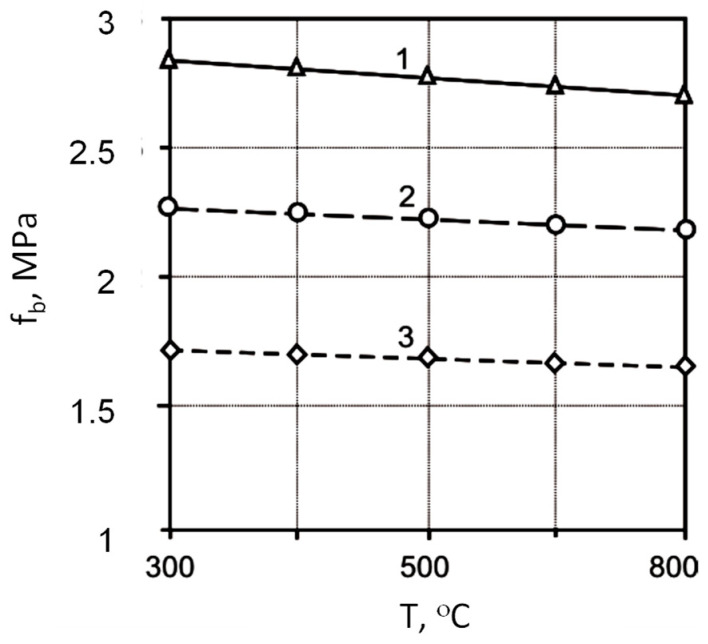
The effect of heating temperature (*T*) and additive consumption (*D*) Na_2_SiF_6_ on the bending strength of mortars (*f_b_*): 1—*D* = 1%; 2—*D* = 0.5%; 3—*D* = 0%.

**Figure 5:**
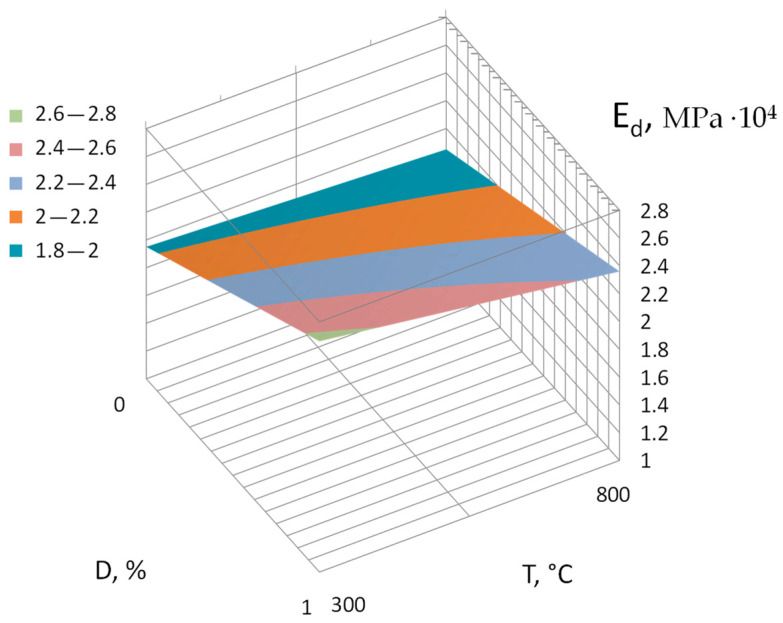
The influence of heating temperature (*T*) and the consumption of the additive (*D*) Na_2_SiF_6_ on the dynamic modulus of elasticity (*E_d_*) of the mortars. The remaining factors were fixed at zero level.

**Figure 6:**
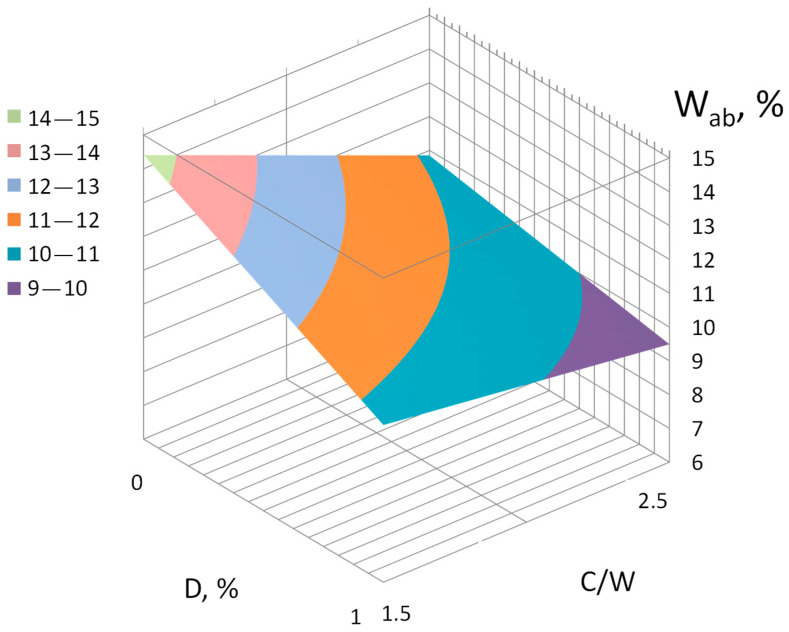
Influence of the C/W and consumption of the additive (*D*) Na_2_SiF_6_ on water absorption (*W_ab_*) of mortars. The remaining factors were fixed at zero level.

**Figure 7:**
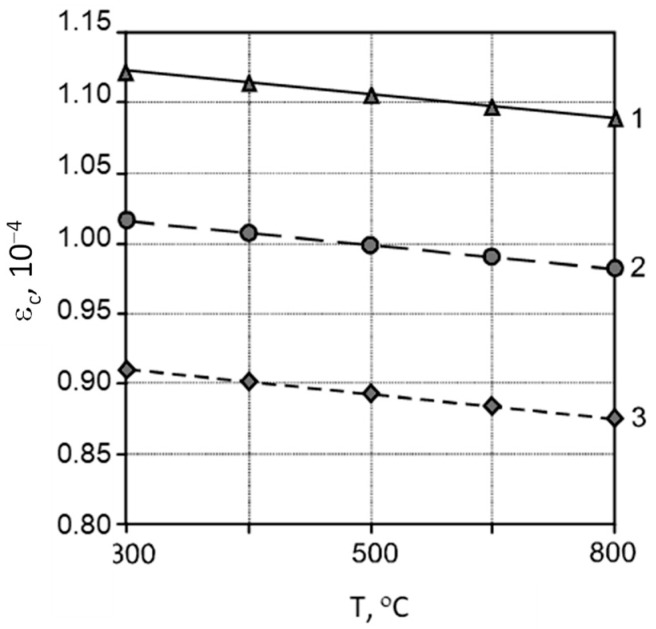
Influence of heating temperature (*T*) and consumption of the additive (*D*) Na_2_SiF_6_ on the conditional extensibility (*ε_c_*) of mortars: 1—*D* = 1%; 2—*D* = 0.5%; 3—*D* = 0%.

The analysis of the obtained regression equations makes it possible to determine the direction and strength of the influence of the studied factors, as well as the effect of their interaction. The application of the Na_2_SiF_6_ additive leads to an increase in compressive and flexural strength, the value of the dynamic modulus of elasticity, conditional elongation, and a decrease in water absorption (Figure 3, Figure 4, Figure 5, Figure 6 and Figure 7).

In addition, the conditional extensibility parameter (3), which characterizes the cracking resistance of concrete, has been calculated [4].

Attention is drawn to the presence in the experimental–statistical models (Formulas (5)–(9)) of certain effects of paired interactions between the factors characterizing the additive content, the cement/water ratio, the heating temperature, and the number of heating and cooling cycles.

To characterize the specific influence of each of the factors on the initial parameter, the coefficient (10) can be used.

(10) $$\chi =\frac{\left|b_{i}+b_{ij}\right|}{b_{0}},$$

where

*b*_0_, *b_i_*, *b_ij_*—regression coefficients of the corresponding regression equations.

Analysis of the values of the coefficient *χ* for indicators of the properties of fly ash concrete after cyclic heating and cooling allows us to rank the factors according to the relative effect of their influence and place them in the following row in descending order: content of (1) Na_2_SiF_6_ additive, (2) cement–water ratio, (3) number of cycles, and (4) temperature. The effect of the first two factors is positive; the others are negative:

- compressive strength *X*_1_ *> X*_2_ *> X*_4_ *> X*_3_;
- bending strength *X*_1_ > *X*_2_ > *X*_4_ > *X*_3_;
- dynamic modulus of elasticity *X*_1_ > *X*_2_ > *X*_3_ > *X*_4_;
- water absorption *X*_1_ > *X*_2_ > *X*_4_ > *X*_3_;
- conditional extensibility *X*_1_ > *X*_4_ > *X*_3_ > *X*_2_.

The analysis of the obtained experimental and statistical models shows that the introduction of the additive and the fluoride activator Na_2_SiF_6_ is an effective technological technique that allows us to preserve and increase the workability of heat-resistant ash-slag concrete under conditions of cyclic heating and cooling regimes.

## 4. Conclusions

The presented research provides the following conclusions:

1. The addition of 20–40% fly ash with 0.5–1% sodium silicofluoride to Portland cement significantly increases the degree of cement hydration at an early stage. The optimal dosage of fly ash for producing heat-resistant mortars is 30% of the cement mass, and for sodium silicofluoride, it is 1%.
2. The developed method of mechanochemical activation, which involves increasing the specific surface area of the binder with the addition of Na_2_SiF_6_, allows for the complete binding of free CaO formed during the decomposition of cement hydration products at a temperature of 800 °C. The effect is enhanced when the specific surface area of the binder is increased to 3500 cm²/g.
3. The use of 30% fly ash by cement mass with an addition of 1% Na₂SiF₆ increases the strength of the fly ash-cement paste by more than twice compared with the strength of plain cement paste when heated to 800 °C. The effectiveness of the fluoride activator further improves as the specific surface area of the fly ash-cement binder increases.
4. After preheating the non-activated fly ash-cement mortar to 600–800 °C, strength loss reached up to 30%. Mechanical–chemical activation of the fly ash-cement binder prevents the reduction in strength after preheating the mortar.
5. The addition of Na_2_SiF_6_ significantly lowers the temperature during deformations of the cement-ash binder in the range of 400 °C to 800 °C.
6. Using the experimental design method, experimental–statistical models of mortar properties were developed, considering composition, heating temperature, and the number of heating–cooling cycles. These models enabled the formulation of quantitative dependencies for predicting mortar properties at elevated temperatures and ranking factors by significance. Optimal values for fly ash dosage, sodium silicofluoride additive, and binder-specific surface area were established. The activator’s positive effect on the thermal deformation of construction mortars was demonstrated.

## Figures and Tables

**Table 1:** Chemical and mineralogical composition of Portland cement.

The content of oxides (%)	SiO_2_	21.5
	A1_2_O_3_	5.19
	Fe_2_O_3_	4.02
	CaO	66.33
	MgO	0.62
	R_2_O	0.28
	SO_3_	1.48
	CaO_f_	0.23
The content of minerals (%)	C_3_S	59.26
	C_2_S	20.05
	C_3_A	6.90
	C_4_AF	11.22

**Table 2:** Physical and mechanical properties of Portland cement.

Specific Surface Area (cm^2^/g)	Normal Consistency (%)	Time of Setting (h-min.)		Strength (MPa)			
		Initial	Final	Flexure		Compression	
				7 Days	28 Days	7 Days	28 Days
2900	25.1	2–30	4–10	4.9	6.5	31.5	52.4

**Table 3:** Conditions for planning experiments when studying the influence of cyclic heating and cooling on the mortar’s properties.

Factor		Levels of Variation		Range of Variation (Intervals)
		−1	+1	
Content additive Na_2_SiF_6_ (D) (%)	X_1_	0	1	1.0
Cement–water ratio (C/W)	X_2_	1.5	2.5	1.0
Heating temperature, (T) (°C)	X_3_	300	800	500
The number of cycles of heating and cooling, (N)	X_4_	15	35	10

Note: Additives were introduced as a percentage of the mass of the cement-ash binder.

**Table 4:** Experiment planning matrix.

No.	Experiment Planning Matrix			
	Additive Na_2_SiF_6_ Content (X_1_ (%))	Cement–Water Ratio (X_2_)	Heating Temperature (X_3_ (°C))	Number of Cycles (X_4_)
1	1	2.5	800	35
2	1	2.5	300	15
3	1	1.5	300	15
4	1	1.5	800	35
5	0	2.5	300	15
6	0	2.5	800	35
7	0	1.5	800	35
8	0	1.5	300	15

**Table 5:** The strength indicators of the activated cement-ash binder obtained by mixing Portland cement and fly ash.

No.	The Composition of the Binder (%)			Strength (MPa) *, at Normal Hardening at Age (Days)						Strength After Steaming ** (T_st_ = 95 °C), After 4 h (MPa)
				Flexural			Compressive			
	Portland Cement (PC)	Fly Ash (FA)	Na_2_SiF_6_	1	3	28	1	3	28	
1	100	-	-	1.74/ 28	3.35/ 54	6.2/ 100	16.2/ 31	23.0/ 44	52.3/ 100	37.1/ 71
2	70	30	-	1.12/ 18	2.48/ 40	4.96/ 80	10.5/ 20	16.7/ 32	40.8/ 78	35.5/ 68
Fly ash is ground to S_sp_ = 3500 cm^2^/g										
3	70	30	0.3	1.43/ 23	3.22/ 52	5.52/ 89	14.1/ 27	20.9/ 40	44.5/ 85	38.2/ 73
4	70	30	0.5	1.67/ 27	3.1/ 50	5.70/ 92	15.7/ 30	23.5/ 45	44.5/ 85	39.2/ 75
5	70	30	1.0	1.74/ 28	3.16/ 51	5.77/ 93	16.7/ 32	24.1/ 46	43.4/ 83	40.3/ 77
Fly ash is not ground (S_sp_ = 2800 cm^2^/g)										
6	70	30	0.3	1.3/ 21	2.91/ 47	5.15/ 83	12.0/ 23	18.3/ 35	42.4/ 81	34.0/ 65
7	70	30	0.5	1.36/ 22	2.98/ 48	5.33/ 86	12.6/ 24	19.3/ 37	42.4/ 81	36.1/ 69
8	70	30	1.0	1.43/ 23	2.98/ 48	5.08/ 82	13.1/ 25	18.3/ 35	42.9/ 82	35.6/ 68

Note: * Before the dash, the strength is in MPa, after—in %. ** Steaming mode, hours: 2 + 3 + 6 + 2.

**Table 6:** The degree of hydration of the cement-ash binder.

No.	The Composition of the Binder (%)			Specific Surface (cm^2^/g)	Hydration Degree During a Period of Observation (Days)		
	PC	FA	Na_2_SiF_6_		1	3	28
1	100	–	–	3500	35	42	55
2	80	20	–	2800	31	43	57
3	80	20	–	3500	33	48	61
4	80	20	–	4200	38	43	68
5	80	20	1	2800	34	48	58
6	80	20	1	3500	37	49	62
7	80	20	1	4200	39	51	69
8	60	30	–	2800	35	45	58
9	60	30	–	3500	37	49	62
10	60	30	–	4200	39	51	63
11	60	30	1	2800	39	52	65
12	60	30	1	3500	41	57	68
13	60	30	1	4200	44	61	72

**Table 7:** CaO_free_ content in cement-ash binder after heating.

No.	Binder Composition (%)	Amount of CaO_f_ After Heating at T in °C (%)			
		500	600	700	800
1	Portland cement 100	8.8	9.5	10.2	10.5
2	Portland cement—70; fly ash—30 (S_sp_ = 2800 cm^2^/g);	8.5	6.1	4.3	2.6
3	Portland cement—70; fly ash—30 (S_sp_ = 2800 cm^2^/g); Na_2_SiF_6_—1	5.6	3.9	2.1	1.5
4	Portland cement—70; fly ash—30 (S_sp_ = 3500 cm^2^/g)	7.1	5.2	2.7	1.4
5	Portland cement—70; fly ash—30 (S_sp_ = 3500 cm^2^/g); Na_2_SiF_6_—1	4.5	2.8	1.4	0.1

Note: Hardening in normal conditions—3 days.

**Table 8:** Relative compressive strength of cement and ash-cement binder when heated (%).

No.	The Composition of the Binder (%)			S_sp_ (cm^2^/g)	Heating Temperature (°C)		
	PC	FA	Na_2_SiF_6_		300	500	800
1	100	-	-	2800	87.5	54.5	30.5
2	70	30	-	2800	72.6	51.3	44.2
3	70	30	1	2800	81.2	64.5	63.8
4	70	30	-	3500	88.8	58.5	50.1
5	70	30	1	3500	89.4	69.9	78.5

Note: 100% is taken as the strength of cement paste under normal hardening conditions.

**Table 9:** Relative linear deformations of heated cement-ash stone.

No.	Binder Composition (%)			Linear Deformations When Heated to Temperatures (°C)		
	PC	FA	Na_2_SiF_6_	400	600	800
1	100	–	–	$\frac{-0.40}{+0.30}$	$\frac{-0.83}{+0.45}$	$\frac{-1.06}{+0.72}$
2	70	30	–	$\frac{-0.25}{+0.20}$	$\frac{-0.70}{+0.40}$	$\frac{-0.75}{+0.58}$
3	70	30	1	$\frac{-0.20}{+0.20}$	$\frac{-0.60}{+0.37}$	$\frac{-0.67}{+0.52}$

Note: 1—“shrinkage”; + “extension”; 2—Na_2_SiF_6_ additive was introduced in the amount of 1% of the mass of cement and ash; 3—ash taken from S_sp_ = 3500 cm^2^/kg.

**Table 10:** Results of implementation of experiments according to plan 2^4−1^.

No.	Strength (MPa)		Dynamic Modulus of Elasticity, E_d_ (MPa∙10^4^)	Water Absorption, W_ab_ (%)	Conditional Extensibility ε_c_
	Compressive f_c_	Bending f_b_			
1	16.83	2.75	2.41	8.61	1.14
2	19.96	3.36	3.21	8.00	1.05
3	11.73	2.57	2.31	12.01	1.11
4	11.07	2.44	2.11	12.10	1.15
5	10.63	2.02	2.20	11.60	0.92
6	9.37	1.69	2.12	12.11	0.74
7	4.33	1.30	1.49	13.80	0.87
8	5.97	1.71	1.79	12.61	0.5

## Data Availability

Raw data were generated at the National University of Water and Environmental Engineering in Ukraine and Cracow University of Technology. Derived data supporting the findings of this study are available from the authors on request.

## Appendix B

**Table A1:** Statistical characteristics of experimental–statistical models calculated using the computer program “PPP”.

	Experimental–Statistical Models									
	f_c_ (Equation (5))		f_b_ (Equation (6))		E_d_ (Equation (7))		W_ab_ (Equation (8))		ε_c_ (Equation (9))	
Standard deviation	0.741		0.561		0.349		0.552		0.228	
Mean quadratic errors	0.262		0.198		0.123		0.199		0.120	
t-criterion (given)	2.02		2.12		2.12		2.12		2.12	
Designation of model coefficients	Value of coefficients	t-criterion (calc.)	Value of coefficients	t-criterion (calc.)	Value of coefficients	t-criterion (calc.)	Value of coefficients	t-criterion (calc.)	Value of coefficients	t-criterion (calc.)
b0	11.24	102.2	2.225	103.0	2.205	127.8	11.350	201.3	0.999	120.5
b1	3.662	33.3	0.55	25.5	0.305	17.7	−1.28	22.6	0.106	12.8
b2	2.962	26.9	0.225	10.4	0.28	16.2	−1.18	20.8	−0.03	3.0
b3	−0.11	1.0	−0.05	2.3	−0.10	5.9	0.05	0.9	−0.02	2.15
b4	−0.83	7.5	−0.18	8.1	−0.17	10.0	0.3	5.3	0.00	0.0
b12	0.537	4.9	0.05	2.3	−0.02	1.2	0.6	10.6	0.02	2.4
b13	−0.26	2.4	0.02	0.9	−0.05	2.7	0.02	0.4	0.00	0.0
b14	−0.11	1.0	0.01	0	0.077	4.5	0.125	2.2	−0.02	2.7
Fisher’s criterions										
Given F_t_	4.45		3.68		3.68		3.68		3.68	
Calculated F_c_	3.81		2.72		3.02		2.86		3.54	
